# IgA and the intestinal microbiota: the importance of being specific

**DOI:** 10.1038/s41385-019-0227-4

**Published:** 2019-11-18

**Authors:** Oliver Pabst, Emma Slack

**Affiliations:** 1 Institute of Molecular Medicine, RWTH Aachen University, Aachen, Germany; 2 Institute of Food, Nutrition and Health, Department of Health Sciences and Technology, ETH Zürich, Zürich, Switzerland

## Abstract

Secretory IgA has long been a divisive molecule. Some immunologists point to the mild phenotype of IgA deficiency to justify ignoring it, while some consider its abundance and evolutionary history as grounds for its importance. Further, there is extensive and growing disagreement over the relative importance of affinity-matured, T cell-dependent IgA vs. “natural” and T cell-independent IgA in both microbiota and infection control. As with all good arguments, there is good data supporting different opinions. Here we revisit longstanding questions in IgA biology. We start the discussion from the question of intestinal IgA antigen specificity and critical definitions regarding IgA induction, specificity, and function. These definitions must then be tessellated with the cellular and molecular pathways shaping IgA responses, and the mechanisms by which IgA functions. On this basis we propose how IgA may contribute to the establishment and maintenance of beneficial interactions with the microbiota.

## Introduction

While scientific progress is rarely straightforward, IgA’s Odyssey seems particular confusing. IgA is the most abundantly produced antibody isotype but was the last isotype to be discovered. Research on IgA biology has dominated the emerging field of mucosal immunology and today we experience an exciting increase in the number of high impact studies on IgA. Nonetheless, we are lacking a comprehensive picture. Extensive work has been performed to study pathways of IgA generation and IgA memory but experts in the field barely agree on the relevance of IgA inductive sites and mechanisms.^1,2^ Counting ourselves as IgA supporters, we suggest that much of the confusion in the field might come from linguistic and biological oversimplifications. Aiming to avoid such inaccuracies, in this review, we will singly discuss new aspects in intestinal secretory IgA (SIgA) biology. We will base our discussion on critical definitions in IgA biology (see also Box 1) and focus on the interaction between SIgA and the intestinal microbiota. For an overview of IgA inductive compartment and class switch recombination please refer to Box 2 ‘Fast facts on SIgA’ and references therein. We emphasize that concepts in intestinal SIgA biology discussed here should not be applied lightheartedly to other mucosal tissues such as lung, eye, and urogenital tract or monomeric IgA predominantly present in serum.

**Fig. 1:**
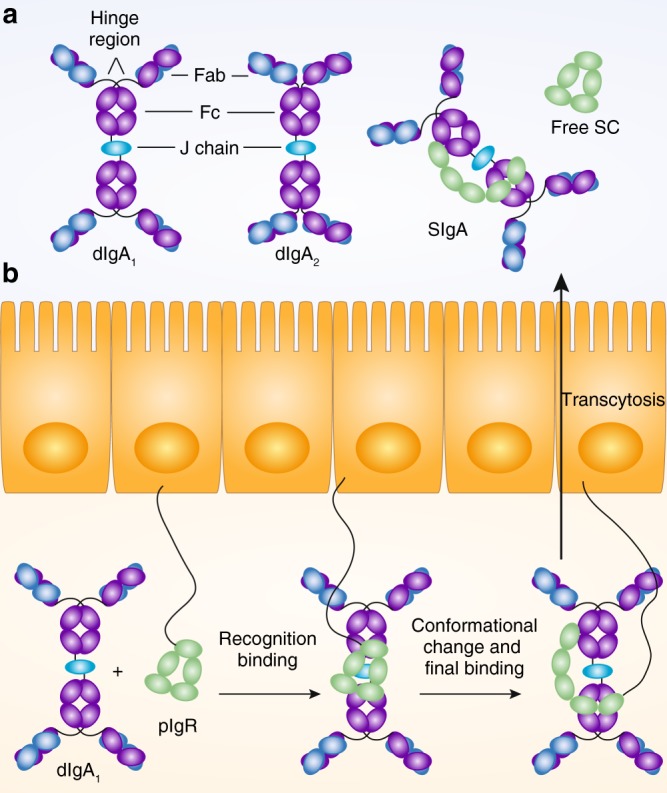
Secretory IgA is formed by the combined function of plasma cells producing multimeric IgA and epithelial cells expressing pIgR **a** Schematic diagrams illustrating the structure of human dimeric IgA1, human dimeric IgA2, human secretory IgA1, and the free secretory component (SC, which is a cleavage product of pIgR). Both, human IgA1 and IgA2 show the canonical antibody structure of two heavy and two light chains building Fab and Fc portions of the antibody. Human IgA1 is characterized by an extended hinge region linking the Fab and Fc part. In dimeric IgA, two antibody monomers are covalently bound through disulfide bonds to the J chain. Secretory component covalently bound to IgA differs in its conformation from free SC. Consequently, free SC and bound SC might have different microbiota binding capacity. **b** Transcytosis of pIgR/dIgA complexes results from initial recognition binding, conformational changes, and final binding before the complex becomes transcytosed. Following transcytosis, free SC, and SIgA are released into the gut lumen (here depicted for human IgA1). Illustrations adapted from refs.^4,68^

IgA shares the archetypical structure of other human and rodent antibody isotypes and is composed of Fab fragments and an Fc region each consisting of several Ig domains (Fig. 1a). However, unlike IgG and IgA in serum, in the human and murine gut, IgA is produced as polymeric IgA (pIgA), foremost as dimeric IgA. The dimeric form of IgA consists of an antibody dimer with two Ig monomers linked tail-to-tail through extensions of the terminal Ig domain of their Fc portion and a protein called joining (J) chain.^3^ Expression of polymeric IgA linked by a J chain is a distinguishing feature of mucosal plasma cells and distinguishes them from plasma cells in many other compartments such as spleen and bone marrow. Thus, the structure of intestinal pIgA is fundamentally different from the prevailing monomeric form of IgA present in human plasma.

The polymeric structure of IgA is a prerequisite for its active transport across mucosal surfaces and secretion. This process is carried out by the polymeric Ig receptor (pIgR). However pIgR does not only transport multimeric IgA but contributes the secretory component that is covalently bound to the antibody portion and constitutes an integral part of the SIgA complex (Fig. 1). Thus, effectively SIgA is a chimeric molecule generated by the combined activity of both plasma cells and pIgR-expressing (mostly epithelial) cells. Consistently, production and secretion of SIgA is not only determined by rates of antibody production by plasma cells but additionally influenced by pIgR expression and activity. Human pIgR is a glycosylated transmembrane protein consisting of five Ig domains forming the pIgR ectodomain also referred to as secretory component (SC), a short transmembrane domain, and an intracellular domain.^3^ During its biosynthesis, pIgR is delivered to the basolateral side of epithelial cells where it binds pIgA (as well as polymeric IgM, not discussed here in more detail). Initial binding is conferred by the first Ig domain, followed by conformational changes in the molecule that further enhance stability of the complex and precede formation of a covalent bond between pIgA and pIgR.^4^ The SIgA complex is transported to the apical side and becomes cleaved to release the mature SIgA. The heavily N-glycosylated SC part of SIgA protects the complex from proteolysis: an essential contribution to its function in the digestive tract. However, free SC is also known to directly interact with intestinal bacteria^5^ and one might speculate that SC as part of the SIgA complex also might confer microbiota binding capacities. Thus, when discussing functions of SIgA, besides the plasma cell produced Fab/Fc portion and J chain, properties of the SC part contributed by epithelial cells have to be considered (Fig. 1).

The pIgR is expressed by various secretory epithelial cells, including those lining the gastrointestinal tract and indeed local production of pIgA by intestinal plasma cells and transport across the gut epithelium contributes most to overall intestinal SIgA production/secretion. Yet, interesting complementation to intestinal SIgA production comes from expression of pIgR in mammary glands that confer transport of SIgA into milk,^6^ and expression in the liver that enables SIgA transport into bile.^7^ While the former pathway is only relevant to passive IgA transfer into very young mammals via milk, the latter pathway, SIgA secretion into bile and thereby the small intestine, also operates in adults. In rodents, polymeric IgA is transported from circulation across pIgR-expressing hepatocytes to the bile canalicular membrane and eventually into bile^7^ enabling the clearance of IgA bound antigen from circulation. In humans, expression of pIgR is restricted to biliary epithelial cells and concentrations of SIgA in bile are lower as compared with rodents. Nonetheless, also in humans IgA is transported into bile and plasma cells producing microbiota directed IgA are present in the liver.^8^ Thus both, SIgA in bile and milk, might create important sources of SIgA that meet particular needs locally in the proximal small intestine and temporally during early life respectively.

Box 1 Critical definitions in IgA biology T-dependent and T-independent SIgA responses: Observations in experimental animal models demonstrate presence of IgA in the absence of T cells. The contribution of T-dependent and T-independent responses to IgA production in mice and human gut is subject of controversy.

Canonical and noncanonical binding of SIgA: Canonical binding describes Fab region-dependent binding of the antibody to its antigen. The antigen specificity of canonical interactions is (mostly) determined by the CDR3 regions. Noncanonical binding describes all other binding modalities, including glycan-dependent binding that are not determined by the CDR3 identity.

Natural and induced SIgA: “Natural” antibodies have a range of definitions in the literature. A common definition is antibodies produced in their germ-line configuration in the absence of exogenous antigenic trigger. However, depending on the point of view, microbiota members are either considered as exogenous antigenic triggers or as endogenous antigens. This should be explicitly stated if using this term. “Induced” responses are described as specific responses to a defined antigen newly entering the system (e.g., a colonizing microorganism or vaccine antigen).

Affinity of SIgA: Affinity describes the strength of interaction between an epitope and an antibody, i.e., the ratio of the rate constant of association and the rate constant of dissociation. Affinity can be determined experimentally only if both antibody and antigen are available in pure form. Affinity of intestinal SIgA for defined epitopes expressed by the microbiota has not yet been determined. As SIgA is dimeric, repetitive antigens may be bound by more than one Fab of the SIgA dimer, yielding a binding avidity.

Polyreactivity of SIgA: Polyreactivity describes the reactivity of an antibody to a variety of structurally unrelated defined antigens such as insulin, LPS, CpG, DNA and others. Polyreactivity is typically linked to natural antibodies produced in germ-line configuration.

Cross-species reactivity of SIgA: We suggest the term ‘cross-species reactivity’ to describe the observation that SIgA complexes binds to seemingly unrelated members of the microbiota. The mechanistic basis of cross-species reactivity is unclear.

Box 2 Immunoglobulin A—fast facts

- IgA is the most abundantly produced antibody in mouse and human. Most IgA-secreting plasma cells are localized in mucosal tissues, foremost the gut.
- In mice a single IgA isotype is present whereas humans have two isotypes, IgA1 and IgA2.^67^
- In mucosal tissues, IgA is mostly produced as dimer linked by the J chain and secreted into mucosal fluids by the polymeric Ig receptor. IgA in serum is mostly present in monomeric form.^68^
- Small intestinal Peyer’s patches are considered main sites of IgA induction (including class switch recombination). Alternative sites such as isolated lymphoid follicles, mesenteric lymph nodes and in situ class switch recombination in the intestinal lamina propria have been described.^2,69^
- Class switch recombination to IgA can occur in the absence of T cells. Mechanisms of T-independent class switch and IgA induction have been reported to include class switch promoting cytokines (e.g., BAFF, APRIL), dendritic cells and innate lymphoid cells.^2,70^
- IgA deficiency is the most prevalent immunodeficiency in humans. IgA deficiency presents with a seemingly mild phenotype, presumably due to the compensation by secretory IgM. Nonetheless, IgA deficiency correlates with predisposition for multiple diseases. Notably, the underlying cause of human IgA deficiency are diverse and some association with disease might not solely be caused by lack of IgA.
- SIgA can protect against toxins and infections.
- IgA can bind to members of the microbiota and affect colonization levels. However, mechanistically, effects of IgA on the microbiota are incompletely understood.

## Critical definitions and nomenclature in SIgA biology

IgA has been called many things. Among others, IgA was suggested to be natural, polyreactive, cross-reactive, primitive, and low affinity. While we appreciate that in the original publications these words will have been chosen very carefully and accurately describe the data, some subtleties will have been lost in follow up work and reviews. A seminal paper published by Andrew MacPherson describing the presence of IgA in T cell-deficient mice may count as an illustrative example. The title of the paper reads ‘A primitive T cell-independent mechanism of intestinal mucosal IgA responses to commensal bacteria’ and in fact, this was the first report to demonstrate generation of IgA by T cell-independent (TI) mechanisms.^9^ MacPherson referred to IgA as induced and not natural because the presence of IgA required the presence of live intestinal microbiota. More recently, another exciting paper published by the Bendelac group reads ‘Natural polyreactive IgA antibodies coat the intestinal microbiota’.^10^ In this report, the authors suggested ‘endogenous mechanism driving homeostatic production of polyreactive IgAs with innate specificity to microbiota’. These papers, published 17 years apart, seem to describe, at the very least, an overlapping family of IgA responses with seemingly contradictory terms.

Clearly this nomenclature can be divided into terms that can be objectively defined (e.g., T-dependent vs. T-independent, canonical vs. noncanonical binding) and those that are subjective based on the system, the type of controls used and the perspective of the authors (primitive, natural, specific, poly/cross-reactive). We will start with the objective definitions and thereafter contemplate the more subjective ones (see also Box 1).

## T-dependent and T-independent SIgA responses to the microbiota

T cell-independent antibody responses are most easily studied in animals that genetically lack T cells, where by definition all antibodies are T cell-independent. However, all antibody responses begin T cell-independently: a naive B cell must first receive an activating signal by antigen-triggered cross-linking of its B cell receptor, typically in the interfollicular/T cell zones of secondary lymphoid structures, or in the subepithelial dome of Peyer’s patches.^11^ This leads to B cell receptor internalization, presentation of BCR-associated antigen on MHCII and migration of B cells to the borders of lymphoid follicles. Typically, at this stage, B cells have the possibility to interact with a T cell, receive help via CD40 and cytokines and enter into a germinal center reaction. In the presence of sufficiently strong cross-linking, as well as alternative costimulatory signals such as Toll-like receptor ligands, these antigen-activated B cells can already undergo proliferation and differentiation into short-lived plasma cells and plasmablasts. However, recent studies in Peyer’s patches suggest that T cell help may already occur during initial B cell expansion in the subepithelial dome.^11^ Plasmablasts typically secrete measurable amounts of specific IgM within a few days of antigen encounter. This response may be replaced by plasma cells leaving germinal centers during the first weeks after antigen exposure. Therefore in T cell-sufficient situations such as wild type mice or humans, T cell-independent responses could also be defined as those occurring very early during a mucosal challenge or where the resulting antibodies have no evidence of having undergone somatic hypermutation, indicating that they are likely the product of this type of response.

It has recently been elegantly demonstrated that intestinal T cell-dependent IgA induction is governed by similar rules as have been observed for systemic IgG production, i.e., that T cell help is the major limiting factor for production of affinity-matured antibody responses.^11^ One exception to this was the observation that T cells were also partially required for the very early stages of B cell expansion in Peyer’s patches.^11^ Correspondingly, gene-targeted mice that overproduced intestinal T follicular helper cells (for example those with a targeted deletion of the extracellular ATP receptor P2X7^12^) display overproduction of specific IgA during intestinal infection or in response to oral vaccination. We may therefore conclude that experimental work in mice clearly demonstrates that T cell-dependent and independent IgA responses can be generated (see also following sections).

## Canonical and noncanonocal binding of SIgA to the micrbiota

We are very used to thinking of canonical Fab-dependent IgA binding to antigens. Canonical, i.e., Fab-dependent binding of SIgA to its antigen, is binding via the complementarity-determining (CDR) regions and adjoining motifs at the end of the Fab arms. In contrast, noncanonical binding is binding via any other (constant) part of the SIgA molecule. The sequence and structure of the CDR3 region can be changed by T cell-dependent somatic hypermutation. Thus, during the process of somatic hypermutation the canonical binding properties of the antibody to the inducing antigen are modified as discussed above. On the contrary, somatic hypermutation does not impact noncanonical binding.

A major contribution to noncanonical binding of SIgA seems to be made not by the amino acids of the constant regions, but by their associated glycans. SIgA is highly glycosylated,^13,14^ with multiple O-glycans attached to each hinge region, 7 N-glycans attached to secretory component and two N-glycans attached to the J chain.

With respect to noncanonical binding of SIgA and the microbiota, glycans in SIgA provide a rich and diverse scaffold for interactions. These glycan structures seem to vary between donors and between the site of SIgA analysis^15^ and may also vary with inflammatory status, rate of IgA production and antibody clone. Thus, determining the contribution of glycan-dependent binding of SIgA to the microbiota is a challenging task. In fact, the nature of noncanonical SIgA glycan–bacteria interaction might differ over time and location in the intestine and noncanonical binding may serve diverse purposes, such as provision of a carbon source,^16^ signals altering bacterial gene expression^17^ or generation or generating weak inter- or intra-species associations.

## “Natural” versus “Induced” SIgA

These terms depend heavily on the type of antibody response being considered. When scientists are looking for a response against a vaccine or infection, then the “induced” response is that observed after the manipulation, and everything pre-existing the manipulation might be considered “natural”. Extending this simple concept to microbiota-targeting immunity, one may only consider antibodies to be “natural” if they are present in a germ-free mouse. If we consider antibodies against components of the microbiota and food-binding antibodies, we may go a step further and only consider antibodies to be “natural” if they are present in an antigen-free mouse. Note that neither of these definitions make any assumptions about T cell help.

IgA is clearly induced by both food antigens and the intestinal microbiota.^18^ However, even during monocolonization of germ-free mice, only a fraction of this induced IgA appears to be microbiota-binding.^18,19^ The remainder may be low-affinity, T-independent antibodies produced by rapidly expanding and amplifying B cells generated early during the response, or may originate from nonspecific amplification of preexisting specificities where we do not know the antigen (potentially microbial antigens not expressed during in vitro culture, environmental antigens, food or self). In fact, fecal IgA levels continuously increase with age in mice, and this is true in both germ-free and colonized animals (albeit with a consistent ten-fold reduction in total IgA levels in germ-free mice^20^). This suggests an age-dependent inducer that may not be live microbial antigens. But is this an increase in “natural” IgA? The answer depends on how “natural” is defined. Potentially also here self, food and environmental antigens may play a role that is experimentally extremely difficult to exclude.

Another commonly-used definition of natural antibodies is that they are germline-like, lacking nontemplated nucleotides and having little to no somatic mutations.^21^ Numerous reports have characterized intestinal IgA by sequence analysis starting in the pre-NGS area to more recent work using Ig repertoire sequencing by NGS methods. Notably, a common observation in these studies is a particularly *high* number of somatic mutations in intestinal biopsies obtained from adult humans.^22^ In contrast, fewer mutations in IgA have been reported in mice. However, comparison of Ig repertoires in young and old mice, as well as in children and in adult humans, indicates that IgA overall is characterized by high mutation rates. Germline-like Ig conformations, in contrast, may represent an unusual phenomenon, mostly present in young mice (such as often used in experimental models) and potentially, in very young children.^23,24^ These findings are consistent between numerous studies and exclude a major contribution of germ-line antibodies to the IgA-secreting plasma cell population in adolescent and adult humans. If mutations in IgA arise over several months, analysis of the commonly used young adult inbred mouse may give a very incomplete picture of the IgA plasma cell population.

An emerging logic is that describing IgA as “natural” is only justified in very specific contexts, and where the authors’ required definition is explicitly stated. In case of vaccination in a normally-colonized animal, microbiota-induced IgA may well be considered “natural” as it is present before vaccination and does not bind to the vaccine antigens prior to vaccination. In contrast, when considering unmanipulated, but colonized mice, SIgA induced by the microbiota does not qualify as natural with the exception of those antibodies that had been present before colonization in an antigen-free context. We therefore strongly caution against using the term ‘natural’ to describe a general property of intestinal IgA unless the term is very carefully defined.

## High versus low affinity binding to the micrbiota

Another important feature of an antibody is its affinity, i.e., the strength of interaction between an epitope and an IgA molecule. Affinity is defined by basic thermodynamic principles governing reversible molecular interactions. Accurately determining affinity, for example by surface plasmon resonance, requires the respective antibody and antigen in pure forms. Currently, no such data have been published that allow for general conclusions about intestinal IgA affinity. Further complications arise from two central tenets of IgA biology: (1) IgA is dimeric, i.e., has four identical binding sites (Note: higher order of polymerization can occur and for instance improves potency of influenza binding is the respiratory mucosa^25^). If the antigen is a repetitive structure, it is possible to have cooperative binding, which increases the avidity of interaction. However, the relationship between avidity and affinity depends heavily on the relative geometry of the repetitive antigen,^26^ the flexibility of the IgA hinge regions, and potentially an unfavorable decrease in entropy associated with restricting movement of both IgA and antigen.^27^ (2) In the case of IgA targeting intestinal bacteria, important surface-exposed antigens are glycans (O-antigens, polysaccharide capsules, and teichoic acids). These are often highly repetitive, but they are also highly diverse,^28^ difficult to obtain in pure form and have high flexibility leading to complex entropy/enthalpy relationships when comparing isolated molecules to molecules in their native densely clustered conformations. It should be noted that there is a huge discrepancy between antibody binding and function. For example, while this has not been extensively studied for SIgA, it is well known that non-neutralizing anti-viral IgG can actually enhance infection.^29^ In addition to classical antigen on- and off-rates, it is useful to have some forms of “functional affinity” readout, representing how SIgA interacts with intact bacteria. For example, where the relevant function is SIgA-driven enchained growth, if the bacterial growth rate and shear-stress on bacteria are known, then it is possible to calculate the mean time that cross-links take to break based on the distribution of bacterial clump size.^30^ In this case, a protective Salmonella-binding polyclonal SIgA response generated bacterial cross-links that break at one quarter of the growth rate. As Salmonella divides roughly every 30 min during the early phase of colonizing in an open intestinal niche,^31^ this corresponded to crosslinks breaking on average after 2 h. Putting numbers on affinities of SIgA interactions, and thus quantifying the energy required to break these interactions, will be a major step forward in understanding the function of different types of SIgA in the near future. Without biophysical measurements of binding we are lacking the grounds to type SIgA interaction as high or low affinity.

## Cross-species reactivity of SIgA

More recently, a new facet of IgA has been discovered. Since long it was known that IgA coats, i.e., binds to the exposed surface of members of the intestinal microbiota. Now a series of articles revealed some surprising properties of this IgA coat. Monoclonal IgA, either classically generated or recombinantly produced, can have a measurable reactivity to a “diverse but defined subset of the microbiota”.^10,32^ This observation seems consistent with other reports and our own unpublished observations. To describe this binding capacity of one monoclonal antibody to bind different members of the microbiota, the term “cross-specificity” has been coined^33^ whereas another publication termed this polyspecificity.^10^ Here we suggest to refer to the phenomenon of a single monoclonal IgA antibody binding multiple unrelated bacterial taxa as cross-species reactivity and will use this term throughout. The term cross-species reactivity avoids the risk to confuse binding to the microbiota with classical polyreactivity (see also Box 2) and does not imply any mechanistic assumption of how this binding pattern comes about. It also emphasizes the key aspect of different (bacterial) species targeted by a single SIgA clone.

**Fig. 2:**
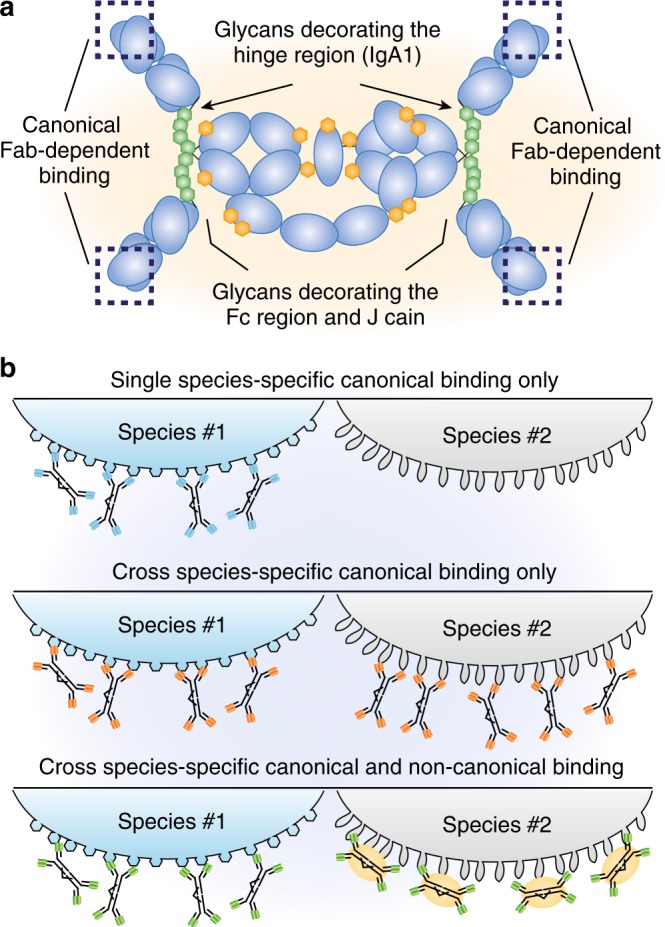
Secretory IgA interacts with the microbiota by canonical Fab-dependent and noncanonical glycan-dependent binding. **a** Schematic diagram of human IgA1 as depicted in Fig. 1. Canonical binding occurs via the Fab regions indicated by dashed boxes. Noncanonical interactions are mediated by glycans. O-Glycans (Yellow-green) are present in the IgA1 hinge region. N-gylcans (red-orange) are present in the Fc portion and the J-chain (modified from ref.^68^) **b** Canonical and noncanonical interactions confer binding to the microbiota. Top panel, schematic illustration of SIgA binding single species in Fab-dependent manner. In this scenario, a given antibody will bind in a highly species-specific manner (here depicted by the antibody binding to one but not another bacterial species). Middle panel, cross-species binding, here depicted by the same antibody binding to two different bacterial species, may occur by canonical interactions if an antibody recognizes epitopes shared between different species. Such binding pattern might occur in case of identical or related shared epitopes expressed by different species. Bottom panel, cross-species reactivity might arise from a combination of canonical and noncanonical binding of antibodies to different bacterial species.

Mechanistically cross-species reactivity has been suggested to correlate with classical polyreactivity, i.e., the ability of an antibody to bind structurally unrelated antigens. The Bendelac group observed that monoclonal IgA antibodies binding to the microbiota, i.e., showing a pattern of cross-species reactivity, were typically polyreactive (bound a set of antigens typically used in polyreactivity assays including LPS, CpG, insulin, and others). Intriguingly, cross-species reactivity was preserved when somatic mutations had been reversed to the germ-line configuration. These observations promoted the authors to suggest that polyreactivity may be a general feature of microbiota-coating IgA. This work represents a tour-de-force in monoclonal antibody generation and prompts further experiments to determine how these observations relate to previous findings. For example, antibodies in germ-line configuration are virtually absent from the human intestinal IgA repertoire (see following section) and it also remains unclear whether cross-species reactive antibodies are targeting structures present in subsets of strains/species (such as common glycan or peptide motifs) or whether cross-species reactivity results from promiscuous binding to a range of different epitopes. These analyses and our understanding of the relevance of these observations would again be hugely advanced by molecular definition of the antigens involved, and quantification of the affinities and modes of binding (Fig. 2).

## Putting recent advances in context

On the basis of the above described definitions (Box 1), we now aim at synthesizing emerging concepts in SIgA biology into a model of how SIgA can modulate the bacterial component of the microbiota. On first sight, some of our definitions might fall into related categories. E.g. T cell-dependent antibodies are typically thought to be induced (e.g., by vaccines or infection) and of high affinity (as measured by surface plasmon resonance). In contrast T cell-independent responses are frequently linked to low affinity antibodies (because they did not undergo affinity maturation to increase their affinity), might have aspects of natural antibodies and have been linked to polyreactivity and cross-species binding to the microbiota.^10^ However, on a second look few of these connections hold up to a more careful inspection.

## SIgA is highly somatically hypermutated: what does this mean?

We start our arguments on a well-documented observation: IgA-secreting plasma cell in the intestine are highly mutated. This is particular obvious in humans^22,34^ but also the plasma cell repertoire of aged mice essentially lacks un-mutated IgA-encoding sequences.^24^ Somatic hypermutation is commonly linked to T cell-dependent germinal center reactions^35^ and constitutive presence of germinal centers is a characteristic of gut associated lymphoid tissue (GALT) of colonized mice. This indicates that intestinal plasma cells might indeed evolve during T cell-dependent responses. On the other hand somatic hypermutation can occur in a T cell-independent manner^36,37^ and Peyer’s patches germinal centers can form independently of cognate B cell receptor signaling and T cells.^38,39^ Thus somatic mutations per se do not exclude a T cell-independent generation of IgA-secreting intestinal plasma cells. In the following section we will weigh arguments to discuss the role of T cell-dependent hypermutation in the generation of SIgA binding to the microbiota. We will not discuss the question of T cell-dependent and T cell-independent class switch recombination to IgA.

T cell deficiency does not completely eradicate SIgA coating of the microbiota. In fact, SIgA coating of only comparably few species, including segmented filamentous bacteria, *Mucispirillum* and *Helicobacter*, was affected by T cell deficiency.^10,32^ This finding demonstrates that SIgA with microbiota-binding capacity can be generated without T cells. However, this finding does not establish that this is indeed the case in a T cell-sufficient situation. The question has been addressed by the Bendelac group by generating a set of recombinant antibodies to represent the IgA antibody repertoire in young (6–12 weeks) mice. Antibodies were expressed as chimeric proteins fusing the IgA derived murine Fab portion to the Fc part of human IgG1. Indeed, in this collection of antibodies, a relevant proportion bound several unrelated members of the microbiota. Since antibodies were expressed as IgG1 fusions, binding to the microbiota was assumed to result from canonical Fab-dependent binding.^10^ Binding to the microbiota was preserved after conversion into the presumed germ-line configuration and the capacity to bind to microbiota was enriched in polyreactive antibodies. Similar binding was observed with broadly neutralizing monoclonal antibodies which bind to influenza HA, suggesting that this type of binding is not unique to intestinal IgA.^10^ These observations prompted the authors to suggest that the majority of species might be coated by SIgA that arises from T cell-independent responses, does not require somatic mutations and binds in germline configuration.^10^ However, nonconventional binding has not been formally excluded. Even though expressed as IgG1 fusions, the antibodies will be glycosylated (IgG1 contains two N-glycans in the Fc portion of the antibody, not dissimilar to N-gylcans found on SC). In fact, variation in glycosylation is a concern in the production of therapeutic human IgG antibodies.^40^ Variable glycosylation is a characteristic feature also of human IgG1 that is highly dependent on the production system, the protein sequence (in particular the rate of acquisition of secondary and tertiary structures during transit through the Golgi), and the rate of production. Thus, these glycans may vary in an antibody-sequence-dependent manner between “identically produced” recombinant monoclonal antibodies.^41^ Potential N-glycan-dependent binding may thus confound our interpretation of binding of the microbiota to in vitro generated antibodies.

Moreover, the exciting concept of natural polyreactive SIgA to coat the microbiota seems at odds with several other interesting reports suggesting that in vivo T cells are required to establish potent SIgA functions. Mice expressing an AID hypomorph capable of mediating class switch but not somatic hypermutation showed hyperplasia of germinal centers in GALT and dybisosis of the microbiota.^42^ This suggests that antigen driven somatic hypermutation of IgA (and potentially IgM) is required to build up potent secretory antibodies. Similarly, T cell transfer into T cell-deficient mice resulted in an IgA-dependent modification of the microbiota and, in a setting enabling the generation of T follicular helper cells, T cell transfer was associated with increased microbiota diversity.^43^ Conversely, CD4+T cell depletion during monocolonization prevented the development of antibodies with sufficient affinity to bind in flow cytometry assays.^44^ These observations indicate that in a wild type setting, somatic hypermutation and T cells are critical to establish a fully functional SIgA system. Further, in accordance with our understanding of T cell-dependent antibody production, the accumulated somatic mutations must have been the result of affinity maturation. Notably, such a scenario is consistent with the high number of somatic mutations observed in intestinal plasma cells.

But what is the function of germ-line like antibodies such as observed by the Bendelac group? We speculate that such germ-line like antibodies might play a particularly important role at young age. Generation of highly mutated SIgA requires time. We observed substantially lower number of somatic mutations in two year old children as compared with adolescents and adult individuals.^23^ Thus germ-like antibodies, many of which exhibit some polyreactivity, might create a first wave of SIgA coating and regulation on the microbiota. Subsequently, the SIgA system might mature to generate more sophisticated antibodies by T cell-dependent somatic hypermutation.

**Fig. 3:**
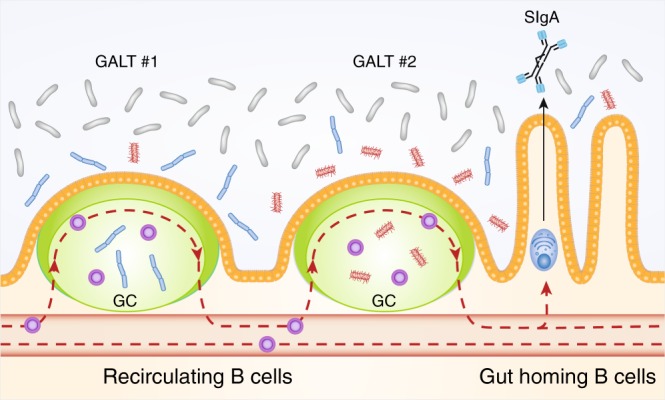
Recirculating B cells coordinate IgA responses in GALT. Antigen experienced B cell are capable of re-entering germinal center (GC). Red dotted lines schematically illustrate B cell migratory routes. GALT#1 and GALT #2 are displayed to represent two different GALT structures such as Peyer’s patches or isolated lymphoid follicles. Different members of the microbiota might have been taken up into GALT and create distinct antigenic environments. We speculate that repeated rounds of selection in different GC, putatively including different antigens, might contribute to the generation of affinity matured cross-species reactive antibodies.

Generation of highly diversified SIgA likely represents a progressive process that entails activated B cells undergoing repeated rounds of selection in germinal centers. The Lycke group observed clonally related B cells in different Peyer’s patches.^45^ Thus, the IgA response might not be formed in a single germinal center but instead recirculating activated or memory B cell might re-enter existing germinal centers (including germinal centers in different GALT, Fig. 3). Consistently, we observed that following intestinal plasma cell depletion within only a few days a new plasma cell population was re-established that largely mirrored the clonal composition before depletion^24^ and memory B cells in Peyer’s patches shared clones also present in the intestinal IgA secreting plasma cell population.^23^ Effectively, such a system of circulating B cells undergoing repeated rounds of stimulation will give rise to highly mutated plasma cells typically observed in the intestinal IgA population. Notably the antigen driving selection must not be identical in different germinal centers. Over time antigen derived from different members of the microbiota might contribute antigen with sufficient affinity to drive B cell selection. Such a system might actively generate cross-species reactive antibodies, as affinity maturation selects for increased affinity for the currently presented antigen without any selection for specificity. Consistent with such mechanisms we observed that in a collection of antibodies generated from human highly mutated intestinal plasma cells, somatic mutations were contributing to cross-species binding (unpublished observations, Kabbert and Pabst). Of course such mechanisms will need to work on the basis of some initial affinity for microbiota antigens present in the primary B cell repertoire.^46^

## The glycobiology of SIgA–microbiota interactions

Owing to the challenging nature of the tools and nomenclature involved, glycobiology is still approached with trepidation by most immunologists. However, it has potential to become the “elephant in the room” in SIgA biology, and is increasingly difficult to ignore. Not only is SIgA heavily glycosylated,^13^ but also the majority of abundant surface-exposed antigens on commensal microbes are glycans (e.g., O-antigens, Teichoic acids/lipoteichoic acids, polysaccharide capsules).^28^

The glycans on IgA have been most closely studied due to their association with IgA nephropathy.^47^ In fact, IgA nephropathy is linked to altered O-glycosylation of the hinge regions of IgA.^48^ It is important to note that there are major structural differences between N- and O-linked glycans: O-glycans (present in the hinge regions, as well as on intestinal mucins) have a wide range of structures; N-glycans (present in SC and the J-chain) all share a core chitobiose attached to three mannose residues, which can be extended by the addition of further mannose, N-acetyl glucosamine, galactose, and sialic acids. These glycans affect SIgA function. Firstly, extensive glycosylation confers protease resistance and IgA without glycosylated secretory component is rapidly degraded in the intestine. Binding of SC N-glycans to bacteria has also been directly linked to innate defense against pathogens,^49^ potentially by competing for binding to bacterial adhesins. In this aspect, it should be noted that binding to soluble IgA will be thermodynamically unfavorable compared to binding to a heavily gylcosylated cell surface, at least in the absence of cooperative binding. A third aspect may in fact be bacteria binding the IgA rather than vice versa. We are now very comfortable with the concept of bacteria foraging mucin-linked O-glycans. It should logically follow that SIgA-linked O- and N-glycans also represent an abundant potential carbon source for the microbiota. Indeed *Bacteroides fragilis* can survive on mammalian N-glycans as a sole carbon source.^50^ Further, a recent study identified that genes with homology to the starch-utilization operon in *Bacteroides thetaiotaomicron* are required for noncanonical interaction with an ovalbumin specific IgA.^17^ Although further molecular work will be required to define the nature of this interaction, it is highly tempting to speculate that *Bacteroides thetaiotaomicron* is actively binding SIgA-associated glycans.

Bacterial glycans as targets of canonical IgA binding pose even greater challenges. Most of what we know about antibody binding and induction comes from very elegant work on small rigid haptens such as nitrophenol,^51^ to which these hydrophilic, long, flexible, repetitive, non-stoichiometrically modified glycans bear little resemblance. NP-specific antibodies are induced in a T cell-dependent way due to the covalently attached protein.^52^ Bacterial glycans such as the O-antigen of lipopolysaccharide are archetypal T-independent antigens.^53^ However, we and others have clearly observed major T-dependent antibody responses to these glycans when they are encountered in the context of whole bacteria.^31,54^ This suggests that co-association of protein antigen in the same cells can be sufficient for T cell help, and defining bacterial glycan-binding antibodies as T-independent per se is not valid.

Whilst the extremely hard work of the glycobiology field has elucidated the structure of many *E. coli, Salmonella* and other pathogenic bacterial surface glycans (e.g.,^55^), we have very little information on most commensal glycans. These are potentially highly diverse, as phage predation tends to drive diversification, for example leading to use of a much broader range of monosaccharides than mammalian glycans.^28^ However, some glycan structures are in fact shared between divergent species. For example the K100 capsule of commensal *E. coli* is almost identical to a capsule of pathogenic *Haemophilus influenzae* giving some degree of natural protection in adult humans with K100 *E. coli* in their microbiota.^56^ Cross-species binding is therefore not necessarily indicative of promiscuous binding: potentially the same glycan motif can be present on two unrelated species. As these glycans are costly to produce, are reversible phage receptors and are functionally important to protect bacterial membranes from attack by hydrophobic toxins, there is a clear benefit to having surface glycans that can be phase-varied or that can be chemically modified based on sugar availabilities and phage presence.^28^ Both will affect IgA binding to a species in ways that will seem stochastic without a deeper understanding of the glycan structure. Many of the next steps in understanding IgA function will require an understanding of the underlying glycobiology.

## Affinity; avidity; cross linkng probabilities, and function

Some mystery still remains as to the function of the grams of SIgA that bind the commensal microbiota and are excreted every day. Suggestions have ranged from promoting colonization of the mucus to neutralization of virulence mechanisms. One of the dominant proposed functions of SIgA is “immune exclusion”: loosely defined as the ability to prevent antigens and microbes from crossing the epithelial barrier. Bacterial clumping by SIgA is a major part of this^57,58^ and the magnitude of SIgA-mediated clumping is sufficient to explain all oral vaccine-mediated protection from nontyphoidal *Salmonella*.^31^ Moreover, in these cases, clumping driven by oral vaccine-induced SIgA was sufficient to drive extinction of a commensal *E.coli* from the microbiota.^31^ This process has also been reported for vancomycin-resistant *Enterococci*.^58^ Therefore SIgA can have sufficient affinity to drive clumping by either classical agglutination or enchained growth of microbiota species. But can endogenous microbiota-binding antibodies, such as those recently described by the Bendelac group achieve this? If we consider the requirements for enchained growth and/or classical agglutination in the context of our above definitions, we can derive the following insights:

T cell dependence of SIgA: For efficient bacterial cross-linking, we need antibody responses with fast association rates and slow dissociation rates, corresponding to affinities in the nanomolar range. In the case of *Salmonella* and *E. coli*, we have not observed any such antibodies to be generated in the absence of T cell help,^31^ suggesting that affinity maturation is essential to generate sufficient affinity. However, we cannot exclude this as a possibility with different bacterial surface antigens that may have a higher affinity for germ-line antibody configurations.

Canonical and noncanonical binding: With respect to generating binding with slow dissociation rates, the possibility of cooperation between canonical and non-canonical binding of IgA could play a major role in stabilizing bacterial–SIgA interactions. However, this has not been extensively analyzed and functional correlates are unclear. In some cases, noncanonical interactions alone may be of sufficient strength and valency to drive cross-linking between two bacteria, for example where bacterial adhesins form catch-bonds to IgA glycans.^59^

Natural and induced SIgA: All observations of efficient bacterial clumping by IgA in vivo have used systems of vaccination or primary and secondary infections, with clump formation typically only observed in “immune” and not in “naive” animals.^57,58^ This suggests that “natural” IgA, using any of the definitions of “natural”, is not sufficient to drive bacterial cross-linking in vivo, at least for all species where this has been currently studied.

Polyreactivity, polyspecificity, and cross-species binding: Another critical determinant of efficient bacterial clumping by SIgA in the gut lumen is the probability that two antigenically identical bacteria come into contact. For bacteria alone in a mixed aqueous environment, this becomes inefficient at densities below 1e8 bacteria per ml.^31^ If you introduce 1e11 other bacteria and a high fluid viscosity, the probabilities that any two identical bacteria to meet become even worse, unless they make up more than around 10% of the total microbiota. Polyreactivity and cross-species binding may therefore serve a purpose to cross-link a targeted species to an abundant cross-reactive microbiota member, allowing classical agglutination to occur despite unfavorable bacterial encounter dynamics. Of course the efficiency of this depends on the strength of the weakest link, such that if the affinity is much lower for the commensal antigen than for the target antigen, this will be inefficient. Conversely, if the affinity is higher for the abundant commensal antigen, competition for binding may result in the rare pathogenic target being spared. The functional consequences of this may be therefore very difficult to generalize.

SIgA-independent factors: Another major consideration in whether an SIgA response will clump a particular bacterial target is the nature of the bacterial antigens being bound. Cross-links can be considered as a system of coupled springs where the overall strength is determined by the weakest link. If the antigen is very weakly associated with the bacterial surface, cross-linking will be inefficient regardless of antibody affinity. Similar problems arise if the antigen is rare or stochastically expressed (i.e., binding partners expressing the antigen are even more rare than the bacterial species/strain). Additional factors that can affect clumping efficiency include the growth rate driving enchained growth and the total bacterial density for classical agglutination, which determine the rate of bacterial encounters.^31,30^

Taken together, whilst bacterial clumping can have major effects on microbiota composition, it is not clear that endogenous SIgA fulfills the requirements to achieve this function.

## SIgA in the context of the intestinal ecosystem

**Fig. 4:**
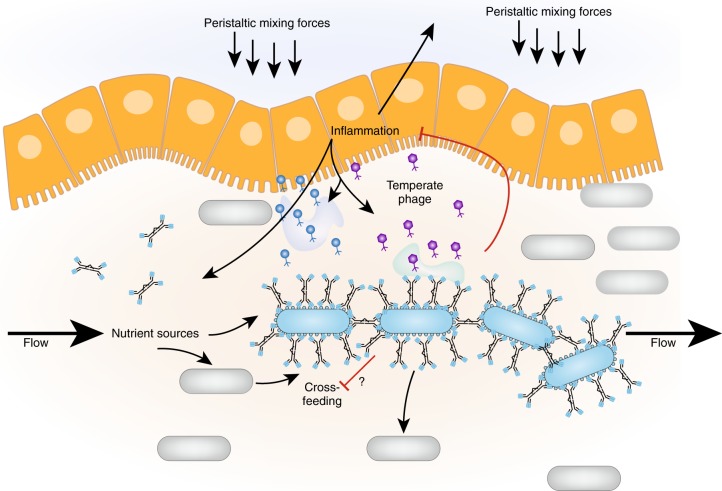
A wide range of selective pressures can be affected by SIgA in the gut lumen. Direct negative selective effects on SIgA-bound bacteria include increased flow-mediated clearance due to enchained growth or agglutination, as well as neutralization of secreted virulence factors. We could also have direct positive effects via metabolism of abundant SIgA O- and N-linked glycans and generation of beneficial community structure by enchained growth/agglutination. Further, extensive indirect effects, i.e., also affected species that do not directly bind SIgA, are expected if the localization or abundance of a metabolic keystone species is altered, altering metabolic network interactions. We also expect major indirect effects due to alterations in the level of immune system activation in the gut due to changes in flow rates, nutrient availability, antimicrobial substances, and phage reactivation.

A critical, and frequently oversimplified aspect of SIgA biology is that unlike serum antibodies, which typically target a single invasive bacterial species in blood or tissues, SIgA works in the context of a densely colonized, flowing, highly-dynamic ecosystem. The environmental conditions (pH, flow rates, nutrient availability, bacteriotoxic compounds etc.) vary dramatically along the intestine length, and typically also fluctuate with food intake and circadian rhythm. Depending on the ecosystem structure, chaotic or robust responses to altering the behavior of a single species could be imagined (Fig. 4).

Particularly dramatic effects are expected if you alter the pro-inflammatory behavior of a single species. Inflammatory effectors in the intestine can directly kill sensitive species,^60^ can alter the range of electron acceptors present,^61,62^ can alter physiological parameters such as flow rate and also generate a dominant signal for the reactivation of temperate phages.^63^ Phage reactivation is expected to have a particularly dramatic effect on overall microbiota composition.^64^ Preventing the pro-inflammatory behavior of a strain, or eliminating a pro-inflammatory strain from the microbiota by SIgA therefore affects not only the targeted species, but potentially the entire microbiota.

At this point it is interesting to note that members of the microbiota that cannot be coated in the absence of T cell-dependent SIgA share a common feature of some degree of invasiveness. Such microbes include segmented filamentous bacteria (SFB),^65^ and *Helicobacter* species.^32^ Further, transfer of IgA-coated bacteria induced more severe colitis as compared to transfer of bacteria not coated with IgA.^32^ Thus, coating with T cell-dependent IgA seems to define preferentially members of the microbiota with enhanced inflammatory potential, and this is likely to play a major role in maintaining the intestinal ecosystem in a state conducive to fostering a diverse, healthy microbiota.

Robust indirect effects could also be expected, for example if the abundance of a keystone species in a metabolic interaction was decreased or increased by the action of SIgA, or if the community structure was altered. This would additionally suggest that functional cross-species reactivity of SIgA should be carefully regulated (i.e., are unlikely to be “natural”) to avoid pathologically disrupting healthy network functions.

In other words, SIgA is only one of many selective pressures acting on bacteria in the intestinal microbiota. Microbiota species can rapidly evolve and will rapidly optimize their fitness in the presence of these selective pressures.^66^ We have clearly evolved an SIgA system that contributes to the control of pathogens and the pathogenic microbiota species by spatially restricting these species, by neutralizing toxins/virulence mechanisms and by driving their local extinction.^31^ This limits or resolves period of inflammation, thus maintaining a healthy gut ecosystem. However, given that only a small fraction of SIgA appears to be carrying out this function, a major question still remains as to what the non-aggregating SIgA is doing. Is adherence to mucus beneficial for the overall luminal population given that the mucus-resident population is smaller by several orders of magnitude? Are SIgA-derived glycans a major carbon source? Can some SIgA response actually increase retention in the gut, rather than promote clearance? These questions still seek mechanistically solid answers.

## Conclusions and open questions

The field of SIgA has recently made major progress, largely driven by the availability of high-throughput sequencing technologies. However, interpreting these data in terms of function still requires further research. We see the following major frontiers as critical to generating a comprehensive model:

1. The contribution of both mammalian and bacterial glycobiology to the generation and function of SIgA. Unfortunately, glycobiology is rarely taught in undergraduate immunology and the technology for glycan research remains highly specialized: two very major barriers to its progress.
2. Appreciation of IgA function at the level of the intestinal ecosystem. This will require development of models that allow quantification of bacterial population dynamics and within-host evolution in a spatially and temporally resolved manner along the intestine. Ideally this should be integrated with better physiological and metabolic analysis of the intestine to quantify indirect vs. direct effects of IgA.
3. Understanding the induction, nature and function of cross-species reactivity, including identification of cross-reactive antigens or noncanonical binding motifs, and quantification of the binding affinities.

With this knowledge, it will finally be possible to apply our now increasingly robust definitions of T-dependent/independent response, canonical- and noncanonical interactions and high/low-affinity interactions to move beyond simple descriptions of binding to elucidate functional mechanisms that are required and that could be detrimental for healthy microbiota function.
